# Design and Synthesis of Hybrid Thermo-Responsive Hydrogels Based on Poly(2-oxazoline) and Gelatin Derivatives

**DOI:** 10.3390/gels8020064

**Published:** 2022-01-18

**Authors:** Annelore Podevyn, Sandra Van Vlierberghe, Peter Dubruel, Richard Hoogenboom

**Affiliations:** 1 Supramolecular Chemistry Group, Centre of Macromolecular Chemistry (CMaC), Department of Organic and Macromolecular Chemistry, Ghent University, Krijgslaan 281, 9000 Ghent, Belgium; annelore.podevyn@gmail.com; 2 Polymer Chemistry and Biomaterials Group (PBM), Centre of Macromolecular Chemistry (CMaC), Department of Organic and Macromolecular Chemistry, Ghent University, Krijgslaan 281, 9000 Ghent, Belgium; sandra.vanvlierberghe@ugent.be (S.V.V.); peter.dubruel@ugent.be (P.D.)

**Keywords:** hybrid hydrogel, poly(2-oxazoline), gelatin, thermo-responsive polymer, amidation

## Abstract

The combination of natural and synthetic polymers to form hybrid hydrogels offers the potential of fabricating new materials that possess a combination of properties resulting from both types of polymer classes. Within this work, two alkene-functionalized poly(2-alkyl/aryl–2-oxazoline) (PAOx) copolymers and one gelatin derivative, thiolated gelatin (gel-SH), are synthesized as precursors for hybrid hydrogels through a photo-induced radical thiol-ene crosslinking process. In-situ photo-rheology revealed an increased mechanical stability for hydrogels that possess an excess amount of PAOx precursor. A final qualitative investigation of the thermo-responsive properties of a P(EtOx_270_–norbornenOx_30_):gel-SH (2:1) hydrogel film revealed a cloud point temperature (T_cp_) in the same range as the T_cp_ of the P(EtOx_270_–norbornenOx_30_) polymer precursor, which is around 30 °C. This promising result demonstrates that thermo-responsive hybrid poly(2-oxazoline)-gelatin hydrogels could be prepared with predictable T_cp_s and that further investigation into this appealing feature might be of interest. Ultimately, this work shows a proof-of-concept of using PAOx as potential hybrid hydrogel precursor in combination with cell-interactive gelatin derivatives to potentially improve the mechanical stability of the final scaffolds and introduce additional features such as thermo-responsiveness for the purpose of drug delivery.

## 1. Introduction

Hybrid hydrogels are hydrogel systems that are composed of a combination of synthetic and natural polymer building blocks, which leads to enhanced material properties and controlled biological functions. Often it is found that synthetic polymer hydrogel scaffolds suffer from the absence of cell-interactive moieties and biological cues that need to be incorporated by polymer functionalization while, conversely, natural polymer-based scaffolds do exhibit these favorable cell-interactive properties but often lack sufficient mechanical stability and strength due to their limited crosslinking density. Therefore, several hybrid hydrogels have already been developed, which combine the mechanical stability of synthetic polymers (i.e., polyethylene glycol, polyvinyl alcohol, (poly(2-oxazoline)s (PAOx), poly(meth)acrylic acid) with cell-interactive properties of natural polymers (i.e., chitosan, hyaluronic acid, gelatin, alginate) [1,2,3,4,5,6].

The use of gelatin for hydrogel synthesis is of great interest in biomedical applications, as this collagen-derived natural polymer possesses a range of appealing advantages, such as an excellent biocompatibility and biodegradability, low cost, cell-adhesive structure due to its RGD-peptide sequence and the availability of a diverse range of functional groups for chemical modifications with crosslinkers, labelling and/or targeting groups. This provides the resulting gelatin-based hydrogel with cell-interactive moieties and biological cues, often required in applications such as tissue engineering [7].

PAOx-based hydrogels are not naturally cell-interactive and require the incorporation of bioactive signaling moieties, such as RGD-peptide sequences, to achieve the desired cell-binding properties [8,9]. Nevertheless, PAOx polymers are biocompatible (including non-cytotoxicity), as is reported for poly(2-methyl–2-oxazoline) (PMeOx) and poly(2-ethyl–2-oxazoline) (PEtOx) [10,11], and their physico-chemical properties and functionality can be tuned in a facile and efficient way through (co)polymerization, side-chain modification and chain-end functionalization, allowing control over the crosslinking density, solubility and mechanical properties. Moreover, a wide range of PAOx hydrogels have been reported using various synthesis approaches [12,13,14,15,16,17,18,19,20,21,22,23]. In this work, a relatively long polymer with a degree of polymerization (DP) of 300 is chosen to achieve sufficient mechanical strength due to chain entanglements. In further research, this might be altered to evaluate the influence of the DP on the physical properties of the final hydrogels.

Here, gelatin and PAOx are used as precursor materials for the development of hybrid hydrogels. To our knowledge, little research has been reported on combining these two materials, limited to N–hydroxysuccinimide (NHS)-ester functionalized PAOx copolymers coated onto a gelatin patch [24,25], physically crosslinked blends of gelatin with poly(ethylene imine) based on hydrogen bonds [26] and covalently crosslinked gelatin hydrogels with short PEtOx chains [27]. This last work reports the effect of using short (DP 50) dialdehyde end-functionalized PEtOx polymers as crosslinking agents for gelatin and revealed an increased swelling behavior with a higher modification percentage attributed to the increased hydrophilicity of the resulting gels [27]. Additionally, the sustained drug release profile and cell viability study showed the potential of using these hybrid hydrogels as scaffolds for drug delivery applications. These promising results encourage further investigation towards combining these building blocks and the versatile chemistry platform of PAOx.

**Figure 1:**
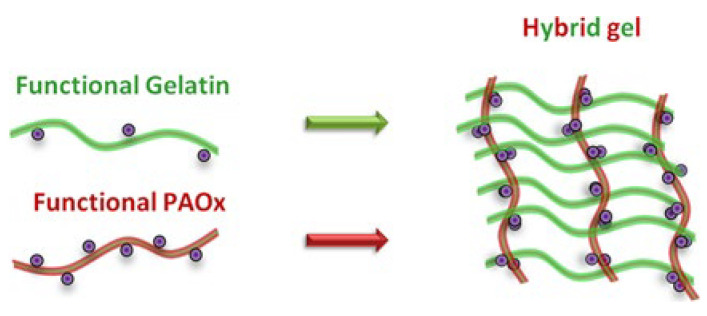
Schematic representation of the formation of a hybrid hydrogel construct composed of thiol-functionalized gelatin and alkene-functionalized PAOx. A stoichiometric balance of functional groups is used for both polymers, which in combination with the lower functionalization degree of the gelatin compared to the PAOx results in a larger amount of gelatin in the final construct.

In this work, the synthesis and physico-chemical characterization of PAOx-gelatin hybrid hydrogels are reported (Figure 1). Initially, the synthesis of the polymer precursors is discussed, as well as their full characterization. Functional PAOx copolymers bearing methyl ester groups as side chains (MestOx) are synthesized and used for a post-modification strategy by direct amidation using allylamine and aminomethyl norbornene, respectively, to effectively introduce crosslinkable groups onto the copolymer. The allyl-amine modified PAOx were recently reported to be efficient precursors for making PAOx hydrogels using dithiothreitol as crosslinker leading to biocompatible hydrogels [28]. Here, thiolated gelatin (gel-SH) was synthesized allowing the formation of hybrid hydrogels by radical thiol-ene cross-linking ensuring the interconnectivity of the PAOx and gelatin precursors. Due to the high reactivity of the thiol-groups towards carbon–carbon unsaturated ‘ene’ functionalities, gel-SH has found numerous uses in the fabrication of (hybrid) hydrogels for tissue engineering [29,30,31,32,33] and drug/gene delivery [34,35,36,37,38], exhibiting good cell encapsulation properties with high cell viability [39,40,41,42].

After developing this small ‘toolbox’ of precursor PAOx and gelatin materials, the hydrogelation kinetics and mechanical properties of several hybrid hydrogel solutions are reported based on photo-rheology. Additionally, hybrid hydrogel films are prepared and evaluated for their swelling properties, structural stability and sol-gel fractions. Finally, a thermo-responsive hydrogel is prepared by making use of the thermo-responsive PAOx precursor, P(EtOx_270_–norbornenOx_30_), and the resulting hydrogels are assessed qualitatively.

## 2. Results and Discussion

### 2.1. Synthesis and Characterization of Polymer Precursors

A first step in the development of hybrid hydrogel scaffolds is the synthesis of photo-crosslinkable building blocks. As primary materials, gelatin and poly(2-alkyl/aryl–2-oxazoline) (PAOx) were chosen, then functionalized with photo-polymerizable groups, such as thiols or alkenes, to allow photo-induced crosslinking between the two classes of described polymers.

#### 2.1.1. Synthesis of Functional Poly(2-oxazoline) Copolymers with Reactive Side Chains

For the synthesis of a functional poly(2-oxazoline) (PAOx) copolymer as starting compound for the functionalization reactions, EtOx and C_3_MestOx (2-methoxycarboxypropyl–2-oxazoline) were chosen as monomers. The methyl ester-containing monomer allows for a straightforward introduction of a wide variety of functional groups in the copolymer side chains through amidation and, therefore, a straightforward tunability of its thermal (solution) properties. C_3_MestOx has been chosen over the C_2_MestOx (2-methoxycarboxyethyl–2-oxazoline) monomer as the literature indicates faster incorporation of the C_3_MestOx monomer in copolymers compared to the C_2_MestOx monomer, which results in a more random-like monomer distribution along the polymer chain upon copolymerization with EtOx [43]. In addition, the copolymer should be water soluble and easily excreted from the body through the kidneys. EtOx is therefore selected as co-monomer because of the hydrophilic character and, relatively, high cloud point temperature (T_cp_ ~ 65 °C) of the resulting PEtOx [44,45,46]. Generally, waste products are removed from the body by filtration of the blood in the kidneys through pores with a size comparable to the hydrodynamic volume of the substances. Considering that the threshold for renal clearance lies around a molecular weight of 30 kDa to 50 kDa for polymers in general and above 40 kDa for PEtOx, we aimed for a copolymer with a DP of around 300, which corresponds to a molecular weight of ± 32 kDa [47,48,49].

**Figure 2:**
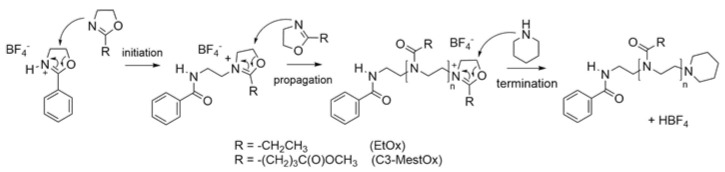
Cationic ring-opening polymerization (CROP) mechanism of EtOx and C_3_MestOx with an oxazolinium salt (2-phenyl–2-oxazolinium tetrafluoroborate, HPhOx–BF_4_) as initiator and piperidine as terminating agent.

In Figure 2, the co-polymerization reaction of EtOx and C_3_MestOx is shown. A simplified literature method was applied, using a 2–phenyl–2-oxazolinium tetrafluoroborate salt (HPhOx–BF_4_) as initiating agent.

**Figure 3:**
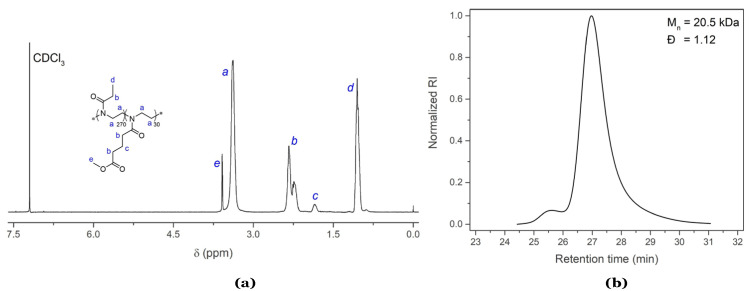
^1^H NMR spectrum (**a**) and SEC eluogram (**b**) of a P(EtOx_270_–*stat*–C_3_MestOx_30_) copolymer with target DP of 300. 2–Phenyl–2-oxazolinium tetrafluoroborate salt (HPhOx–BF_4_) and piperidine were used as initiating and terminating agent, respectively.

For the synthesis of well-defined high molar mass PEtOx [50], all glassware was silanized prior to the polymerization to eliminate trace amounts of water that could lead to premature termination of the polymer and, therefore, an increase in the polymer molar mass distribution, i.e., dispersity (Ð). The HPhOx–BF_4_ initiator was molten under deep vacuum before adding the monomers and solvent (CH_3_CN) to ensure a uniform initiation step and even polymer chain growth. The copolymerization was performed at 60 °C as low temperature reduces chain transfer reactions during propagation [51]. Finally, piperidine was used as terminating agent instead of the more standard methanolic KOH solution, to avoid saponification of the labile methyl esters. Isolation of the pure copolymer was performed by precipitation in diethyl ether followed by dialysis against distilled water and lyophilization, resulting in a white, fluffy powder. ^1^H NMR spectroscopy and size exclusion chromatography (SEC) results of the purified P(EtOx_270_–*stat*–C_3_MestOx_30_) copolymer product are shown in Figure 3 revealing a number average molecular weight (M_n_) of 20.5 kDa and a low dispersity (Ð) value of 1.12. The off-target molecular weight can be attributed to the fact that the M_n_ was calculated relative to PEtOx standards. A small high molecular weight shoulder is observed which results from unavoidable chain transfer reactions and subsequent chain coupling often observed in PAOx synthesis [52,53,54]. The ^1^H NMR spectrum shows all the characteristic peaks for both EtOx and C_3_MestOx repeat units, confirming the theoretical monomer ratio.

#### 2.1.2. Side-Chain Modification via Direct Amidation

**Figure 4:**
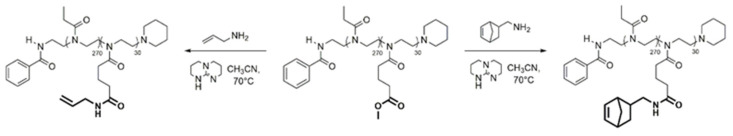
Post-polymerization modification of the methyl ester side groups of P(EtOx_270_–*stat*–C_3_MestOx_30_) with allylamine (left) or aminomethyl norbornene (right) in CH_3_CN at 70 °C, using 0.5 equivalents of TBD [55].

Post-polymerization modification of P(EtOx_270_–*stat*–C_3_MestOx_30_) with allylamine or aminomethyl norbornene results in a functional PAOx copolymer with reactive double bonds in the side-chain that can be used in the preparation of hybrid hydrogels, as described later in this paper. In both modification strategies, the P(EtOx_270_–*stat*–C_3_MestOx_30_) copolymer was dissolved in CH_3_CN and reacted with the respective amine at 70 °C in the presence of TBD as catalyst, as shown in Figure 4.

**Figure 5:**
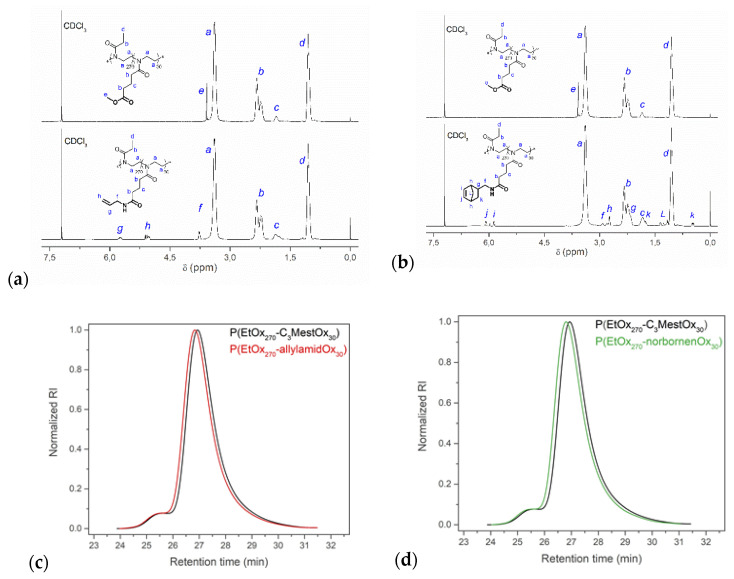
Above; ^1^H NMR spectra of P(EtOx_270_–*stat*–C_3_MestOx_30_) before and after complete modification with allylamine (**a**) or aminomethyl norbornene (**b**). Below; SEC eluograms of P(EtOx_270_–*stat*–C_3_MestOx_30_) before and after complete modification with allylamine (**c**) or aminomethyl norbornene (**d**), i.e., respectively P(EtOx_270_–allylamidOx_30_) and P(EtOx_270_–norbornenOx_30_) for simplification.

^1^H NMR spectroscopy was used to monitor the conversion of the methyl ester groups to the corresponding amide groups. The spectra before and after modification (Figure 5a,b) show the disappearance of the methyl ester peak (3.6 ppm) and the appearance of the corresponding allylamide or norbornene-amide peaks, indicating near-quantitative conversion. After work-up, the purified polymers were obtained as white powders and stored in a dry and inert atmosphere. The SEC traces, shown in Figure 5c,d, illustrate a nearly identical distribution before and after modification, indicating the absence of polymer coupling side reactions during post-polymerization amidation of the side chains.

**Figure 6:**
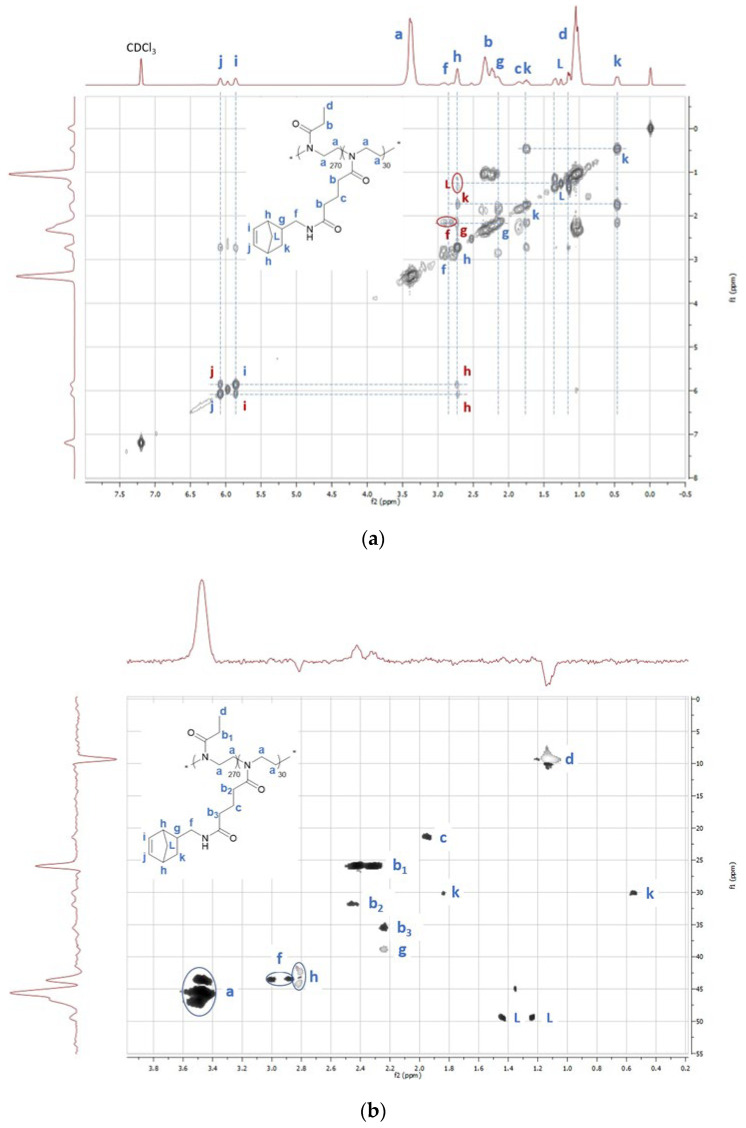
^1^H–^1^H COSY (**a**) and ^1^H-^13^C HSQC (**b**) 2D spectra of P(EtOx_270_–norbornenOx_30_), used for the correct assignment of the peaks in the ^1^H NMR spectrum of Figure 4. A zoomed-in HSQC spectrum is used for clarity, excluding the cross-peaks for protons “i” and “j” that lie around 135 ppm in the ^13^C spectrum.

To correctly assign each of the norbornene-amide peaks in the proton spectrum, ^1^H-^1^H COSY and ^1^H-^13^C HSQC 2D NMR spectra were recorded and examined as shown in Figure 6. Homonuclear correlation spectroscopy (^1^H-^1^H COSY) was used to identify the different proton signals (*f-L*) of the norbornene unit via coupling with each other. The alkene protons *j* and *i* were identified due to their higher chemical shift and show a clear correlation. Their correlation with the adjacent *h*-protons allows identification of the peak at 2.73 ppm. These *h*-protons, in turn, have a link with the protons *g*, *k* and *L*. Identification of the protons of *g* could be based on their correlation with the *f* methylene protons at 2.8–2.9 ppm, but defining the protons for *k* and *L* was more difficult. Therefore, a ^1^H-^13^C heteronuclear single quantum coherence (HSQC) spectrum was recorded to gain information on the correlation between two different nuclei separated by a single bond. The x-axis depicts the ^1^H NMR spectrum while the y-axis shows the ^13^C NMR spectra allowing differentiation between *k* and *L* as the carbon atom of *L* exhibits a higher chemical shift (49.5ppm) than the carbon atom of *k* (30.1 ppm) [56].

#### 2.1.3. Influence of the Side-Chain Modification on the Thermo-Responsive Behavior

**Figure 7:**
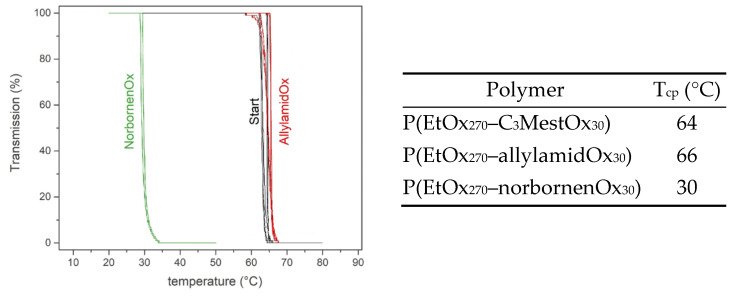
Overview of the cloud point temperatures (T_cp_) determined for the P(EtOx_270_–*stat*–C_3_MestOx_30_) copolymer (i.e., “start”) and the polymers obtained after modification with allylamine (i.e., allylamidOx) and aminomethyl norbornene (i.e., norbornenOx). All T_cp_s were measured in deionized water with a polymer concentration of 10 mg/mL.

Upon preparation of both functional PAOx copolymers, the effect of the side-chain modifications on the thermo-responsive properties of the resulting polymer was explored by determining the T_cp_s at a polymer concentration of 10 mg/mL in deionized water (Figure 7).

The solubility of the polymers was, expectedly, found to depend on the polarity of the copolymer. The starting copolymer possesses a T_cp_ of 64 °C. Modification with allylamine increased only slightly the T_cp_ to 66 °C, which is presumably due to the competing influence of an increase in chain length and the presence of the double bonds as well as the polar amide groups. The modification with aminomethyl norbornene led to a significant drop in T_cp_ to 30 °C. This could be expected as the norbornene units are relatively large and nonpolar, causing the functionalized polymer to be less hydrophilic. As seen from this small data set, the T_cp_ of the starting copolymer can be easily fine-tuned over a broad temperature range by making use of a direct amidation modification with different amines. Additionally, the norbornene-functionalized copolymer possesses a T_cp_ in between room temperature and body temperature which may provide further functionality in biomedical applications such as drug delivery, immunotherapy, bio-imaging or tissue engineering [57,58,59].

#### 2.1.4. Development of Thiolated Gelatin (Gel-SH)

**Figure 8:**
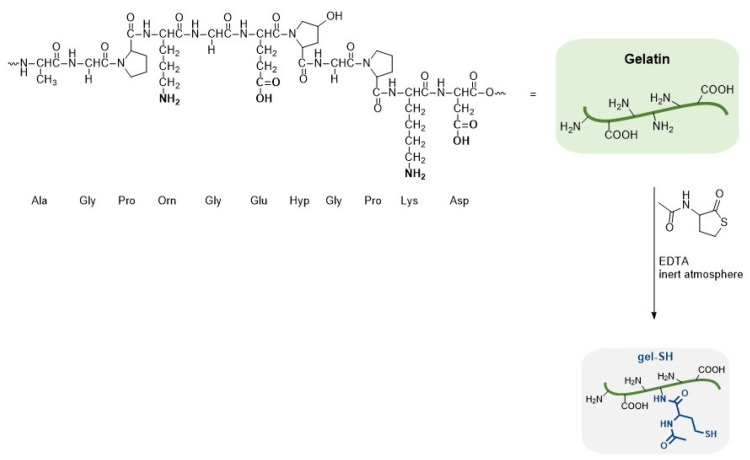
General chemical structure of gelatin and its modification reaction for preparation of thiolated gelatin (gel-SH).

For the development of the second base material, gelatin was functionalized by modification of the primary amines with *N*-acetylhomocysteine thiolactone at 40 °C, resulting in thiolated gelatin (i.e., gel-SH), as depicted in Figure 8. The modification strategy for the synthesis of thiolated gelatin type B was based on a previously reported aminolysis reaction with *N*-acetylhomocysteine thiolactone [60,61]. The reaction was performed at 40 °C to completely dissolve the gelatin and convert the primary amine groups of lysine, hydroxylysine and ornithine to sulfhydryl-terminated groups. As thiolated groups cannot be detected unambiguously by ^1^H NMR spectroscopy, an alternative technique is used for the calculation of the DS of gel-SH based on a spectrophotometric assay with ortho-phthalic dialdehyde (OPA), as described in the Methods section. In this indirect method, the amount of free amine groups in gelatin before and after modification are determined by reaction with OPA and 2-mercaptoethanol. This reaction results in the formation of an isoindole chromophore which exhibits a maximum absorbance at 335 nm. By comparing the difference in absorbance before and after modification, the unreacted amount of free amine groups can be determined, thereby enabling the calculation of the degree of substitution (DS) of gel-SH. The addition of 5 equivalents of *N*-acetylhomocysteine thiolactone (relative to the primary amines) resulted in a DS of 56% (batch 1) or 63% (batch 2).

### 2.2. Investigation of the Crosslinking Kinetics and Mechanical Properties of PAOx:gel-SH Hybrid Hydrogels via Photo-Rheology

**Figure 9:**
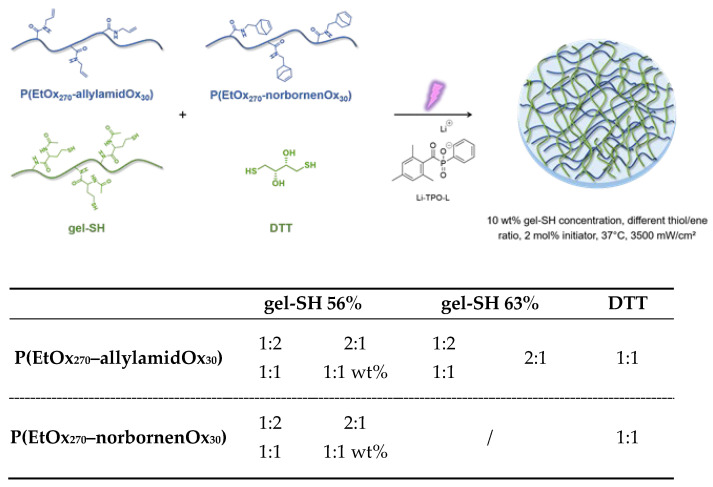
Overview of the different hydrogel solutions, containing Li–TPO–L as photo-initiator, a synthetic precursor (PAOx, vertical column) and thiolated crosslinker (gelatin or DTT, horizontal row), used to determine the crosslinking kinetics of the hybrid gels. For each combination of the precursors, the measured molar ratios of PAOx to gelatin are given as well.

After successful synthesis of the potential hydrogel precursors, the focus was shifted towards the preparation of the hybrid hydrogels. Within this paper, only the mechanical properties were investigated as a proof-of-concept study to assess the influence of each of the precursors on the stability and strength of the final hybrid hydrogel scaffolds. For this, initial tests were performed using photo-rheology to study the gelation kinetics of different hydrogel precursor solutions containing P(EtOx_270_–allylamidOx_30_), P(EtOx_270_–norbornenOx_30_) together with gel-SH with a DS of 56% and 63% or 1,4-dithiothreitol (DTT) (Figure 9). Hydrogels with DTT were used as a reference in which the mechanical strength could solely be ascribed to the crosslinked PAOx precursor. As photo-initiator, lithium (2,4,6–trimethylbenzoyl)phenylphosphinate (Li–TPO–L) was used instead of the more standard Irgacure 2959, because of its higher solubility in water and its increased reactivity at 365 nm (relevant for future cell encapsulation) due to its absorption maximum around 375 nm. Furthermore, the Li–TPO–L initiator exhibits a higher efficiency of radical formation and a larger addition rate constant to double bonds, making it a superior candidate for crosslinking of these hybrid hydrogels [62]. The crosslinking kinetics of the different precursor solutions were determined as follows. In brief, an aqueous solution of gel-SH (10 wt%) was heated to 40 °C in order to exceed its upper critical solution temperature (UCST) and become fully soluble. Upon complete dissolution, the functionalized PAOx polymer was added in different ratios with respect to the functional groups (1:2 mol%, 1:1 mol%, 2:1 mol% or 1:1 wt%, Figure 9). In case DTT was used, a 1:1 molar ratio mixture was prepared with a 10 wt% functionalized PAOx polymer concentration in deionized water. Finally, Li–TPO–L was added and a 100 µL sample was taken for measurement on the rheometer. The samples were irradiated with UV-A light (365 nm) at 37 °C and were measured within the linear visco-elastic (LVE) region to prevent causing any structural damage to the gels. After complete crosslinking of the gel, the measurement continued for an additional 5 min without UV light to test the post-curing of the formed hydrogel.

**Figure 10:**
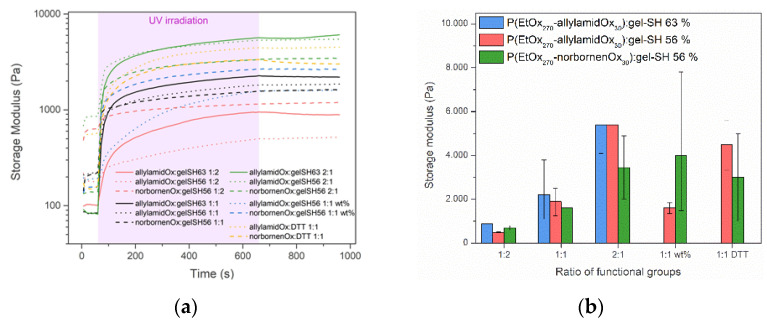
Comparison of the storage modulus of hybrid hydrogel solutions upon crosslinking at 37 °C with UV light at 3500 mW, obtained by photo-rheology (average of 2 measurements). The hydrogel solutions contain 10 wt% gel-SH as natural polymer and P(EtOx_270_–allylamidOx_30_) or P(EtOx_270_–norbornenOx_30_) as synthetic polymer, in 4 different ratios. Additional measurements with 1,4–dithiothreitol (DTT) instead of gel-SH were also performed. Li–TPO–L was used as photo-initiator in all experiments. (**a**) The data as obtained by the rheology measurement. (**b**) A comparison of the final storage modulus values, reached after reaching a plateau value.

The photo-rheology results depicted in Figure 10 showed a steep increase of the storage modulus (G′) upon UV irradiation, indicating the high reactivity of the photo-initiator which causes almost immediate crosslinking and complete formation of the hydrogel for all PAOx:gelatin or PAOx:DTT combinations. Furthermore, an increased G′ was observed when more PAOx precursor was incorporated in the gel (i.e., 2:1 mol% > 1:1 mol% > 1:2 mol%) indicating the formation of a stronger hydrogel. This might be explained by the formation of additional crosslinking points by coupling of the excess of double bonds present from the functionalized PAOx [63]. These results nicely demonstrate that the synthetic PAOx polymer can be used to increase the mechanical stability of the final hybrid hydrogel with gel-SH. It should be noted that a larger error is found on the results of the 1:1 wt% gels, as this correlates to a ∼ 6.5:1 mol% mixture that was much more viscous and not as homogeneous compared to the other solutions. Furthermore, the increased mechanical stability of the PAOx polymers is also reflected in the hydrogels containing only PAOx and DTT as thiol agent, indicating that the initial mechanical stability can be ascribed to the PAOx precursor.

### 2.3. Determination of the Mechanical and Swelling Properties of PAOx-Gelatin Hybrid Hydrogel Films

A deeper investigation into the physico-chemical properties of the hybrid hydrogel films, consisting of P(EtOx_270_–allylamidOx_30_) and gel-SH (DS 56%) was performed by determining the mechanical strength, swelling properties and gel fraction of the macroscopic hydrogel films. Preparation of the hydrogel films was done by dissolving gel-SH in deionized water at 40 °C (above its UCST) and adding P(EtOx_270_–allylamidOx_30_) to the solution in a 1:1 mol% ratio with respect to the functional groups. Upon complete dissolution, Li–TPO–L was added as photo-initiator and the mixture was injected into a mold with a thickness of 1 mm and irradiated for 30 min from both sides to obtain a uniform, crosslinked film. From this, disks with a diameter of 14 mm could be cut out for further characterization of the hydrogel sheet.

#### 2.3.1. Mechanical Properties of PAOx-Gelatin Hybrid Hydrogel Films

**Figure 11:**
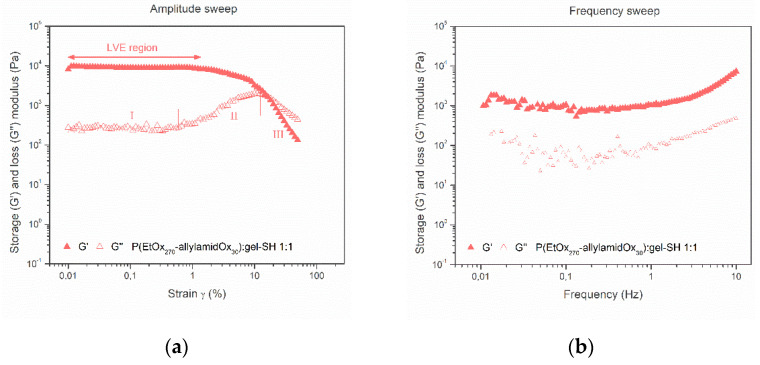
Amplitude sweep at 1 Hz frequency (**a**) and frequency sweep (**b**) for hybrid hydrogel films (1 mm × Ø 14 mm) containing P(EtOx_270_–allylamidOx_30_) and gel-SH as photo-crosslinkable precursors. The amplitude sweep data can be divided into 3 main parts (I, II and III), as discussed in the text.

In a first test, the mechanical properties of the hydrogels were determined by means of rheology. The hydrogel disks were incubated in deionized water at 37 °C for 24 h to reach equilibrium swelling before measurement. Initially, an amplitude sweep test was performed at a frequency of 1 Hz to determine the non-destructive deformation range, also called the linear visco-elastic (LVE) region for the 1 Hz frequency that is applied, and critical strain of the hydrogels (Figure 11a). A first look at this data confirmed the gel-like structure of these systems as the G′ values are much higher compared to the loss modulus (G″). At low deformation, the G′ of both hydrogels remain constant (plateau value, LVE region), indicating that the sample structure is still intact and the hydrogel experiences no irreversible damage. Upon increasing the amplitude of deformation, the sample structure is destroyed, which is reflected in a decrease of the storage modulus.

The data (G′ and G″) obtained for the hybrid hydrogels at 1 Hz frequency in Figure 11a can be divided into three main parts. In the first part, at low deformations (I), G′ > G″ which is indicative for gel-like structures and both curves remain at a constant value. At larger deformations (II), G′ still remains at its plateau value and the G″ curve starts to incline, meaning that part of the deformation energy is lost by internal friction. Hence, this part shows that micro-cracks start to develop in the gel structures while the overall gel structure still remains intact. Finally, in the third part of the data plot (III), the G′ curve starts to decline, leaving its plateau value and hence the LVE region. At this point, the G″ curve crosses the G′ curve (G″ > G′) and starts a steep decline, showing that larger fractions disrupt the hydrogel structure. The rather sharp decline of the G′ curve also indicates that the hydrogel undergoes a rather brittle fracturing and breaks into larger, inhomogeneous pieces.

Subsequently, frequency sweeps were performed in the LVE region of the hydrogels (γ = 0.1%, controlled deformation, determined through the amplitude sweep measurement at 1 Hz frequency) to monitor the storage modulus and reveal insight into the materials’ frequency dependency (Figure 11b). High frequencies are used to simulate the short-time behavior (rapid motion), while the low frequency range is used to mimic the long-term behavior or the behavior at rest. The PAOx-gel-SH hybrid hydrogels possess a consistent solid-like structure (G′ > G″) over the complete frequency range and a slight increase of the elastic storage modulus towards higher frequencies was observed. This can be ascribed to the presence of solvents, entanglements and network defects in the hydrogel structure, which become more important at short timescales as the measured modulus no longer represents the equilibrium gel response. The PAOx-gel-SH hybrid hydrogel was found to have a G′ around 1 kPa.

#### 2.3.2. Swelling Behavior and Gel Fraction of PAOx-Gelatin Hybrid Hydrogel Films

**Figure 12:**
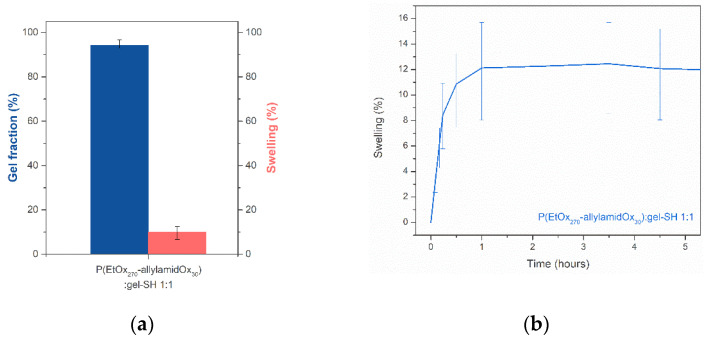
(**a**) Determination of the gel fraction and swelling degree (%) after 24 h of the hydrogels, based on Equations 1 and 2. Each measurement was performed in triplicate; (**b**) Swelling kinetics were performed in deionized water at 37 °C and also performed in triplicate. The standard deviations were marked with error bars.

A second set of tests was performed by looking at the gel fraction and the swelling behavior of the prepared hybrid hydrogels. The resulting data in Figure 12a show a gel fraction above 80% for the PAOx:gel-SH hybrid hydrogels, indicating a good network integrity. Furthermore, the equilibrium swelling degree (after 24 h) of the gel is rather low, indicating a high network density. The swelling kinetics depicted in Figure 12b indicated that the PAOx:gel-SH hydrogels swelled non-uniformly. This was further visually confirmed that the gels did not keep their disk-like shape.

### 2.4. Thermo-Responsive Properties of PAOx:gel-SH Hybrid Hydrogels

**Figure 13:**
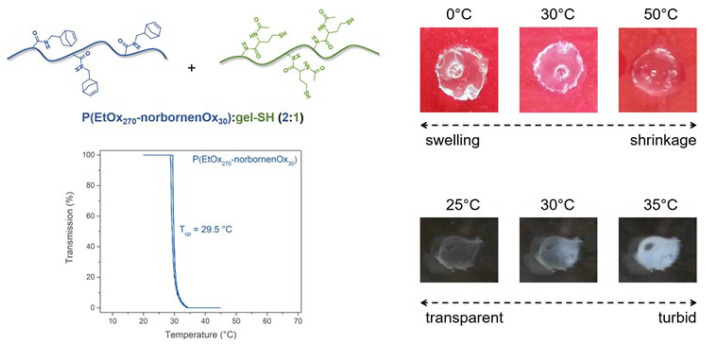
Qualitative observation of the thermo-responsive properties of a hybrid hydrogel consisting of P(EtOx_270_–norbornenOx_30_) and gel-SH in a 2:1 ratio.

In the last section of this paper, P(EtOx_270_–norbornenOx_30_) was investigated as precursor material for the preparation of thermo-responsive hybrid hydrogels, which is another feature that can be added to the hybrid hydrogel constructs by using PAOx polymers as building blocks. To test this, a hybrid hydrogel film containing P(EtOx_270_–norbornenOx_30_) and gel-SH (DS 56%) as precursor materials in a 2:1 ratio and Li–-TPO–L as photo-initiator was prepared as described above. The resulting hydrogel was washed to remove any unreacted species and dried overnight in a 70 °C oven. Subsequently, the thermo-responsive properties of this hydrogel were determined by incubating the gel in deionized water and monitoring the change, qualitatively, over a temperature profile between 25 °C and 35 °C. This temperature profile was chosen as the P(EtOx_270_–norbornenOx_30_) precursor exhibits a T_cp_ of 30 °C at 10 mg/mL concentration in deionized water. Figure 13 shows a similar change in transparency around 30 °C for the hybrid hydrogel, indicating that the T_cp_ of the PAOx precursor material can be directly translated to the T_cp_ of the resulting hybrid hydrogel.

Future work might explore this feature more in-depth and determine the properties of the thermo-responsive gel in a more quantitative way. Nevertheless, this initial test already shows the potential of P(EtOx_270_–norbornenOx_30_) as a building block for thermo-responsive hybrid hydrogels.

## 3. Conclusions

Hybrid hydrogels consisting of synthetic and natural polymers aim to combine the properties of both precursor types to create a gel with superior properties compared to their single polymeric hydrogel counterparts. In this paper, the mechanical stability (and thermo-responsive properties) of the synthetic PAOx polymers are combined with the cell-interactive properties of the natural gelatin polymer. Interestingly, the use of Li–TPO–L as a highly reactive photo-initiator leads to a very efficient curing reaction with almost immediate crosslinking of the PAOX-gelatin hydrogels. Overall, comparing the different hydrogels with varying ratios of both precursor materials led to the conclusion that mechanically stronger gels were formed (higher G′) when more PAOx polymers (i.e., alkene units) were incorporated. Finally, by using DTT as reference thiolating agent, it could be demonstrated that the strength of the hybrid hydrogels is mainly ascribed to the presence of PAOx, since the PAOx:DTT hydrogels had a similar strength compared to PAOx:gel-SH hydrogels. In the final part of this paper, the potential of creating thermo-responsive hybrid hydrogels by using a thermo-responsive P(EtOx_270_–norbornenOx_30_) building block was qualitatively examined via a temperature screening of the resulting hybrid hydrogel film with gel-SH. Subjecting the hydrogel film to a temperature change around the T_cp_ of P(EtOx_270_–norbornenOx_30_), which is 30 °C, demonstrated a similar change in transparency around the same temperature. Further investigations would be required to determine this thermo-responsive behavior quantitatively and explore the potential of varying the T_cp_ by changes in ratios and/or concentrations of the thermo-responsive precursor. Additionally, its potential for an orthogonal crosslinking mechanism with thiolated gelatin allows for the synthesis of homogeneous hybrid hydrogel networks. Further investigation into the biocompatibility of these gels could be tested through cell studies, while degradation studies can provide information on the degradation time in function of the crosslinking degree and/or hydrogel composition. Moreover, tissue constructs with locally tunable mechanical properties and stiffness gradients can be developed and assessed for their potential to support cell proliferation and differentiation of stem cells into various lineages. This will allow us to gain fundamental insight into the correlation between mechanical property variations and cell response.

## 4. Materials and Methods

### 4.1. Materials

Gelatin type B was isolated from bovine skin by an alkaline hydrolysis process and was kindly donated by Rousselot (Ghent, Belgium). All the following materials were used as received unless stated otherwise. Chlorotrimethylsilane (TMS-Cl), 1.5.7–triazabicyclo[4.4.0]dec–5–ene (TBD, 98%), allylamine (≥98%), methacrylic anhydride, sodium hydrogenphosphate (Na_2_HPO_4_), sodium bicarbonate (NaHCO_3_), sodium carbonate (Na_2_CO_3_, anhydrous), potassium dihydrogen phosphate (KH_2_PO_4_), ethylenediaminetetraacetic acid (EDTA), 2-mercaptoethanol, *N*-acetylhomocysteine thiolactone and HPLC grade solvents such as acetonitrile (CH_3_CN, 99.8%) and diethyl ether (Et_2_O) were purchased from Sigma-Aldrich. Aminomethyl norbornene and dithiothreitol were bought from TCI Europe. Barium oxide (BaO, 90%), magnesium sulfate (MgSO_4_, anhydrous, 97%) and ortho-phthalic dialdehyde (OPA) were purchased from Acros Organics. Lithium phenyl–2,4,6–trimethylbenzoylphosphinate (Li–TPO–L) was synthesized according to the procedure reported elsewhere [62]. 2–ethyl–2-oxazoline (EtOx) was kindly donated by Polymer Chemistry Innovations (PCI), distilled over BaO and ninhydrin prior to use and stored in a glove box under inert and dry conditions. C_3_MestOx was synthesized via the modified Wenker method, as described in literature [43]. 2–Phenyl–2-oxazolinium tetrafluoroborate (HPhOx–BF_4_) was synthesized according to the literature procedure [50]. Piperidine was distilled over CaH_2_ prior to use. Dry solvents were obtained from a solvent purification system from J.C. Meyer, with aluminum oxide drying columns and a nitrogen flow. Deuterated solvent for ^1^H NMR spectroscopy, i.e., chloroform-*d* (CDCl_3_, ≥ 99.8% D, water < 0.01%), was purchased from Eurisotop.

### 4.2. Equipment

Monomers and polymerization mixtures were stored and prepared in a Vigor Sci-Lab SG 1200/750 glove box system with water and oxygen levels below 1 ppm. Polymerization samples were measured with gas chromatography (GC) to determine the monomer conversion based on the ratio of the integrals from the monomer and the reaction solvent. GC was performed on an Agilent Technologies 7890A system equipped with a VWR Carrier-160 hydrogen generator and an Agilent Technologies HP-5 column of 30 m length and 0.320 mm diameter. An FID detector was used and the inlet was set to 250 °C with a split injection of ratio 25:1. Hydrogen was used as a carrier gas at a flow rate of 2 mL/min. The oven temperature was increased with 20°C/min from 50 °C to 120 °C, followed by a heating ramp of 50°C/min from 120 °C to 300 °C. Size exclusion chromatography (SEC) was performed on an Agilent 1260-series HPLC system equipped with a 1260 online degasser, a 1260 ISO-pump, a 1260 automatic liquid sampler (ALS), a thermostatted column compartment (TCC) at 50 °C equipped with two PLgel 5 µm mixed-D columns and a precolumn in series, a 1260 diode array detector (DAD) and a 1260 refractive index detector (RID). The used eluent was *N,N*-dimethylacetamide (DMA) containing 50 mM of LiCl at a flow rate of 0.5 mL/min. The SEC eluograms were analyzed using the Agilent Chemstation software with the GPC add on. Molar mass values and dispersity (Ð) values were calculated against PMMA standards from PSS. Deionized water (milliQ) was prepared with a resistivity less than 18.2 MΩ × cm using an Arium 611 from Sartorius with the Sartopore 2 150 (0.45 + 0.2 µm pore size) cartridge filter. Preparative SEC was performed on disposable PD-10 desalting columns from GE Healthcare, following the gravity protocol described in the accompanying instructions. Lyophilisation was performed on a Martin Christ freeze-dryer, model Alpha 2-4 LSC plus. Nuclear magnetic resonance (NMR) spectra were recorded on a Bruker Avance 400 MHz (1D) or 500 MHz (2D, gelatin) spectrometer at room temperature or 40 °C (gelatin) with the chemical shifts (δ) given in parts per million (ppm) relative to trimethylsilane (TMS) or residual solvent signals. Cloud point temperatures were determined using a Crystal16^TM^ parallel crystallizer turbidimeter developed by Avantium Technologies connected to a recirculation chiller and dry compressed air. Aqueous solutions were prepared in deionized water at 10 mg/mL and heated within different temperature ranges depending on the polymer. For all measurements, three heating and cooling cycles were performed with a heating/cooling rate of 0.5 °C/min and a stirring rate of 700 rpm. The cloud point temperatures are given as the 50% transmittance points. UV-VIS spectra were recorded on a Varian Cary 100 Bio UV-VIS spectrophotometer equipped with a Cary temperature and stir control. Samples were measured in either quartz or disposable cuvettes with a path length of 1.0 cm in the wavelength range of 200 to 700 nm. The concentration of each sample was 1.0 mg/mL in deionized water. Rheology measurements were carried out on a rheometer type Physica MCR-301 from Anton Paar with parallel plate geometry and an upper plate diameter of 25 mm. For monitoring the crosslinking reactions, 300 µL solution was placed between the plates using a gap setting of 0.3 mm. The edges were trimmed and sealed using low viscous silicone grease (GE Bayer Silicones) to prevent sample drying. An oscillation frequency of 1 Hz and a strain of 0.1% were applied as these values are within the linear visco-elastic region as determined by isothermal measurements (37 °C) of the storage (G′) and loss (G″) moduli as a function of deformation at a constant frequency (1 Hz) and varying strain (0.01% to 10%). After 1 min measurement, the samples were irradiated at 37 °C using UV-A light (EXFO Novacure 2000 UV light source at 365 nm using an intensity of 3500 mW) for 10 min followed by monitoring the post-curing process for 5 min. Rheology on hydrogel films was performed by placing uniform gelatin disks (14 mm diameter, ± 1 mm thickness) that are swollen to equilibrium (deionized water, 37 °C, 24 h) in between the spindle (25 mm diameter) and the bottom plate of the rheometer at 37 °C. The spindle was lowered with 25 µm increments until a normal force of around 0.6–1 N was observed to ensure proper contact. The storage modulus (G′) was measured at 37 °C using an amplitude of 0.1% over a frequency range of 0.01 Hz to 10 Hz.

### 4.3. Methods

#### 4.3.1. Copolymerization of C_3_MestOx and EtOx

EtOx is copolymerized with 10 mol% C_3_MestOx using a simplified literature procedure [50,64]. All glassware was cleaned and dried in a 200 °C oven, before being silanized with chlorotrimethylsilane (TMS-Cl) to exclude any water from the reaction that might lead to premature termination of the polymer chains and therefore an increase in polymer dispersity. Next, a 2–phenyl–2-oxazolinium tetrafluoroborate salt (HPhOx–BF_4_) (0.003 eq., 303.15 mg, 1.29 mmol) was added to the flask as initiator and melted under active vacuum (1.6 × 10^−1^ mbar). The silanized flask was transferred under an inert and dry atmosphere to a glove box, where the monomers, EtOx (0.9 eq, 39.25 mL, 388.9 mmol) and C_3_MestOx (0.1 eq., 6.43 mL, 43.2 mmol), and the solvent (CH_3_CN, 44.33 mL) were added. The mixture was stirred firmly and a t0 sample was taken as starting point to follow the conversion via GC and ^1^H NMR spectroscopy. To obtain a P(EtOx–C_3_MestOx) copolymer with a target DP of 300 at 91.5% conversion, the reaction mixture was put in an oil bath at 60 °C for 62 h. After the reaction, 0.25 mL of piperidine was added at 0 °C and stirred overnight. Purification was performed by precipitation of the copolymer in ice-cold Et_2_O followed by dialysis (3.5 kDa MWCO) and subsequent lyophilization to obtain the P(EtOx–C_3_MestOx) as a white, fluffy powder (28.7 g, M_n_ = 20.5 kDa, Ð = 1.12).

#### 4.3.2. Post-Polymerization Modification of P(EtOx_270_–stat–C_3_MestOx_30_) by Direct Amidation with Allylamine

The synthesized P(EtOx–C_3_MestOx) copolymer contains 10 mol% (30 units) of methyl ester side chains which were functionalized in a post-polymerization modification step by direct amidation with allylamine. The obtained P(EtOx–C_3_MestOx) copolymer (2 g, 0.0719 mmol), containing 2.156 mmol of functional methyl ester groups (1 eq.), was dissolved in 9.01 mL of acetonitrile and 0.5 equivalents of TBD (150.05 mg, 1.078 mmol) was added. Subsequently, allylamine (6 eq., 0.97 mL, 12.94 mmol) was added and the mixture was degassed by purging with argon and reacted at 70 °C for 30 h to full conversion. The pure polymer was obtained by precipitation in ice-cold Et_2_O followed by dialysis (1000 Da MWCO) and subsequent lyophilization (M_n_ = 21.4 kDa, Ð = 1.19).

#### 4.3.3. Post-Polymerization Modification of P(EtOx_270_–stat–C_3_MestOx_30_) by Direct Amidation with Aminomethyl Norbornene

The P(EtOx–C_3_MestOx) copolymer (2 g, 0.0719 mmol) containing 10 mol% (30 units) of methyl ester side chains (2.156 mmol) was dissolved in 9.01 mL of acetonitrile, after which 0.5 equivalents of TBD (150.05 mg, 1.078 mmol) and 6 equivalents of aminomethyl norbornene (6 eq., 1.595 mL, 12.94 mmol) were added, followed by degassing the mixture with argon. The polymer was modified to full conversion by refluxing the solution for 24 h at 70 °C. Purification of the resulting polymer was done by precipitation in ice-cold Et_2_O, dialysis (1000 Da MWCO) and final lyophilization (M_n_ = 23.0 kDa, Ð = 1.12).

#### 4.3.4. Synthesis of Thiolated Gelatin B (Gel-SH)

Thiolated gelatin was synthesized according to a literature procedure [61]. Under inert atmosphere and firm stirring, 50 g of gelatin B (19.5 mmol amines) was dissolved in 500 mL carbonate buffer (pH 10) (NaHCO_3_ + Na_2_CO_3_ in deionized water) at 40 °C. After complete dissolution, 1.5 mM ethylenediaminetetraacetic acid (EDTA, 219.18 mg, 0.75 mmol) was added in order to complex any metals present in the solution, thereby preventing oxidation of the thiol groups to disulfide bonds [65]. Subsequently, 5 equivalents of *N*-acetylhomocysteine thiolactone (15.52 g, 97.5 mmol), relative to the primary amines, were added and the mixture was left to react for 3 h at 40 °C. Afterwards, the solution was diluted with 500 mL deionized water and dialyzed against distilled water at 40 °C (12–14 kDa MWCO). Finally, the thiolated gelatin was isolated by freezing in liquid nitrogen and successive lyophilization, before being stored at −80 °C to prolongate its shelf life. The entire procedure including the purification was performed under an inert atmosphere to prevent disulfide formation by redox reaction with oxygen. Additionally, a spectrophotometric assay was used to determine the degree of substitution (DS) of the developed thiolated gelatin. For this, a first stock solution was prepared containing orthophthalic dialdehyde (OPA, 20 mg) dissolved in 10 mL ethanol and diluted with 40 mL double distilled water. A second stock solution contained 25 µL 2-mercaptoethanol which was added to 50 mL borate buffer (0.1 M, pH 10). Subsequently, a reference sample was prepared containing 0.5 mL OPA, 1 mL double distilled water and 1.5 mL 2-mercaptoethanol. This sample was used to obtain a calibration plot by comparing it to a series of samples containing a 50 µL n-butylamine standard solution with varying concentration (i.e., 0.002 M, 0.006 M and 0.01 M) in 0.95 mL double distilled water, 1.5 mL 2-mercaptoethanol solution (stock solution 2) and 0.5 mL OPA solution (stock solution 1). For this, the absorbance of the samples was measured at 335 nm and the R^2^ was calculated. After calibration, two solutions were prepared (25 mg/mL in double distilled water), one of the unmodified gelatin B and one of the thiolated gelatin, to compare the difference in absorbance at 335 nm. For the measurements, 50 µL of the gelatin solutions were mixed with 0.95 mL double distilled water, 1.5 mL 2-mercaptoethanol solution (stock 2) and 0.5 mL OPA solution (stock 1), before being submitted to the UV-VIS spectrometer. All measurements were performed in triplicate at 37 °C.

#### 4.3.5. Development of Hybrid Hydrogels Using Functional Gelatin and Functional PAOx via Film Casting

Thiolated gelatin (10 wt%, 1 g, 0.216 mmol functional groups, 56% DS) was dissolved in 10 mL deionized water at 40 °C. After complete dissolution, functionalized PAOx (i.e., P(EtOx_270_–allylamidOx_30_)) was added in a 1:1 molar ratio (153.69 mg) with respect to the functional groups and the mixture was shaken firmly. Finally, 2 mol% (relative to the amount of double bonds) of Li–TPO–L photo-initiator was added and the heated solution was injected between two parallel glass plates covered with a thin Teflon release sheet, separated by a 1 mm thick silicone spacer. The mold was irradiated for 30 min from both sides with UV-A light (365 nm, 4 mW/cm^2^) to obtain a homogeneous hybrid hydrogel film. From this film (±1 mm thick), uniform disks could be cut out (14 mm diameter) for further characterization. The mechanical properties of the hydrogels were determined by rheology where we monitored the storage modulus (G′) at 37 °C, using an amplitude of 0.1% over a frequency range going from 0.01 Hz to 10 Hz.

### 4.4. Characterization

#### 4.4.1. Determination of the Crosslinking Kinetics via Photo-Rheology

Thiolated gelatin (10 wt%, 30 mg, 0.0073 mmol (63% DS) or 0.0065 mmol (56%) functional groups) was dissolved in 300 µL deionized water at 40 °C under vigorous stirring in a dark room. After complete dissolution, functionalized PAOx (i.e., P(EtOx_270_–allylamidOx_30_) or P(EtOx_270_–norbornenOx_30_)) was added in different ratios with respect to the functional groups (1:1 mol%, 1:2 mol%, 2:1 mol% or 1:1 wt%). Finally, 2 mol% (relative to the amount of double bonds) of Li–TPO–L photo-initiator was added (via stock solution) just before measurement on the rheometer. The photo-rheology measurements were carried out at 37 °C, irradiating the sample for 10 min with UV-A light and an additional 5 min without light to evaluate post-curing of the hydrogel. An oscillation frequency of 1 Hz and a strain of 0.1% were used to be within the range of linear visco-elasticity.

#### 4.4.2. Determination of the Gel Fraction and Swelling Capacity of the Hydrogels

Determination of the gel fraction and the equilibrium swelling degree was done by freeze-drying the gels immediately after curing, to remove the water, and subsequent weighing (W_d0_). Next, the films were immersed in deionized water at 37 °C and were allowed to swell for 24 h. After equilibrium swelling, the swollen mass was determined by gently dipping the excess of water of the films and weighing them again (W_he_). Finally, the gels were freeze-dried again and the final dry mass was determined (W_de_). From this, the gel fraction and equilibrium swelling degree could be calculated using Equation (1) and Equation (2), respectively. All measurements were performed in triplicate.

(1) $$\mathrm{Gel}\;\mathrm{fraction}\;\left(\%\right)=\frac{W_{\mathrm{de}}}{W_{d0}}\cdot 100$$

(2) $$\mathrm{Equilibrium}\;\mathrm{swelling}\;\mathrm{degree}\;\left(\%\right)=\frac{W_{\mathrm{he}}-W_{d0}}{W_{d0}}\cdot 100$$

Swelling tests were performed by incubating the gels in deionized water at 37 °C to achieve equilibrium swelling. The hydrogels were then subsequently air dried and dried in a 70 °C oven overnight to obtain the dry weight (W_d0_). The gels were again submerged in deionized water and the swelling degree was measured after regular time intervals (W_ht_). The degree of swelling was calculated using Equation (3). All measurements were performed in triplicate and the standard deviations were marked with error bars in the swelling plot.

(3) $$\mathrm{Swelling}\;\left(\%\right)=\frac{W_{\mathrm{ht}}-W_{d0}}{W_{d0}}\cdot 100$$

## Data Availability

Raw data are available from the authors upon request.
